# RT Prepare: a radiation therapist-delivered intervention reduces psychological distress in women with breast cancer referred for radiotherapy

**DOI:** 10.1038/s41416-018-0112-z

**Published:** 2018-06-01

**Authors:** Georgia Halkett, Moira O’Connor, Michael Jefford, Sanchia Aranda, Susan Merchant, Nigel Spry, Robert Kane, Thérèse Shaw, David Youens, Rachael Moorin, Penelope Schofield, on behalf of the RT Prepare project team

**Affiliations:** 1 School of Nursing, Midwifery and Paramedicine, Faculty of Health Sciences, Curtin University, Perth, WA Australia; 2 School of Psychology, Faculty of Health Sciences, Curtin University, Perth, WA Australia; 3 Department of Cancer Experiences Research, Peter MacCallum Cancer Centre, Melbourne, VIC Australia; 4 Sir Peter MacCallum Department of Oncology, Faculty of Medicine, Dentistry and Health Sciences, The University of Melbourne, Melbourne, VIC Australia; 5 Cancer Council Australia, Sydney, NSW Australia; 6 School of Health Sciences, University of Melbourne, Melbourne, Victoria Australia; 7 Department of Radiation Oncology, Royal Adelaide Hospital, Adelaide, SA Australia; 8 Department of Radiation Oncology, Sir Charles Gairdner Hospital, Perth, WA Australia; 9 Radiation Oncology, Genesis Cancer Care, Perth, WA Australia; 10 Telethon Kids Institute, University of Western Australia, Perth, WA Australia; 11 School of Public Health, Faculty of Health Sciences, Curtin University, Perth, WA Australia; 12 Centre for Health Services Research, School of Population and Global Health, University of Western Australia, Crawley, WA Australia; 13 Department of Psychology, Swinburne University of Technology, Hawthorn, VIC Australia

**Keywords:** Patient education, Quality of life

## Abstract

**Background:**

The aims of this study were to determine whether a radiation therapist-led patient education intervention (RT Prepare) reduced breasts cancer patients’ psychological distress (primary endpoint); anxiety, depression and concerns about radiotherapy, and increased knowledge of radiotherapy and preparedness (secondary endpoints). Patient health system usage and costs were also assessed.

**Methods:**

A multiple-baseline study across three sites. The RT Prepare intervention comprised two consultations with a radiation therapist: prior to treatment planning and on the first day of treatment. Radiation therapists focused on providing sensory and procedural information and addressing patients’ pre-treatment anxiety. Usual care data were collected prior to intervention commencement. Data collection occurred: after meeting their radiation oncologist, prior to treatment planning, first day of treatment and after treatment completion. Multilevel mixed effects regression models were used.

**Results:**

In total, 218 usual care and 190 intervention patients participated. Compared with usual care, intervention participants reported lower psychological distress at treatment commencement (*p* = 0.01); lower concerns about radiotherapy (*p* < 0.01); higher patient knowledge (*p* < 0.001); higher preparedness for procedural concerns (*p* < 0.001) and higher preparedness for sensory-psychological concerns at treatment planning (*p* < 0.001). Mean within-trial costs per patient were estimated at $AU159 (US$120); mean ongoing costs at $AU35 (US$26).

**Conclusion:**

The RT Prepare intervention was effective in reducing breast cancer patients’ psychological distress and preparing patients for treatment. This intervention provides an opportunity for radiation therapists to extend their role into providing patients with information and support prior to treatment to reduce psychological distress.

## Introduction

Approximately 50% of patients experience heightened anxiety and distress prior to radiotherapy^1–3^. Patient information needs are high before treatment planning and treatment commencement^3–5^. Patients require information about planning and treatment procedures, pain or discomfort they might experience and side effects. Information is provided by radiation oncologists, radiation oncology nurses and radiation therapists (RTs); however, information provision is inconsistent and often late^6^. Furthermore, information provided may contain medical jargon that is misunderstood and does not meet patients’ current information needs^7^. In addition, pre-treatment anxiety is rarely considered^8^.

Evidence suggests that currently the psychosocial needs of patients with cancer are not adequately met^9^ and therefore psychological morbidity is underrecognised and undertreated^10^. Untreated comorbid psychological conditions can be detrimental to patients emotionally and physically^11^ and also lead to higher medical costs and longer hospital stays^12^. A longitudinal study with breast cancer patients highlighted that anxiety and depression levels did not change from radiotherapy planning to treatment commencement^3^. Patients who are inadequately prepared for radiotherapy and anxious may not adhere to treatment and take longer to treat on a daily basis^13^. Furthermore, in some cases inadequate information and communication may lead to people declining treatment that might otherwise improve their chance of survival^14^. Appropriate and timely information prior to treatment commencement may reduce distress and increase patient satisfaction^15^. Although studies have developed educational resources^16–19^ and trialled group education^20,21^, the focus has been on general information provision rather than patients’ needs at specific timepoints, or addressing anxiety prior to commencing radiotherapy. Research is required on interventions targeting patient preparation and anxiety; and implementing effective interventions into practice.

Level I evidence on preparing patients for threatening medical procedures indicates that sensory and procedural information, and techniques for addressing anxiety are effective^22–24^. Sensory information focuses on how patients may feel before, during and after the procedure; procedural information describes the procedure. Preparation incorporating these components is more effective in reducing anxiety than other interventions^22–26^. Health professionals require communication skills training to learn the skills to delivery sensory and procedural information and support patients appropriately. Communication skills training focusing on eliciting and responding to patients emotional cues has been demonstrated to improve health professionals’ communication and patient interviewing skills, attitudes towards communicating with patients and health professionals’ confidence in their ability to communicate^27–29^. This combined evidence has not been applied in radiotherapy to improve patient preparation for planning and treatment.

This team developed the RT-led educational intervention RT Prepare, consisting of two one-on-one patient education sessions with an RT prior to planning and treatment commencement. A pilot randomised controlled trial (RCT) was conducted at a single site with 122 participants. The intervention was clinically feasible, effective and acceptable to health professionals and patients^30^. Compared with usual care, intervention participants reported lower anxiety (*p* = 0.048, effect size = 0.29), lower concerns about radiotherapy (*p* = 0.001, effect size = 0.62) and higher knowledge (*p* < 0.001, effect size = 1.16) at treatment planning^31^.

### Aims

This trial aimed to examine the effectiveness of RT Prepare to reduce patient psychological distress before treatment. Psychological distress was measured using the total score for the Hospital Anxiety and Depression Scale (HADs-T), which adds anxiety and depression scores together to provide a score for psychological distress^32,33^. Secondary aims were to examine the effectiveness of the intervention to: reduce patient anxiety and depression; reduce concerns about radiotherapy; increase patient knowledge of radiotherapy and increase preparedness for planning and treatment. Patient health system usage and costs were assessed.

### Primary hypothesis

After controlling for baseline distress, intervention patients will report significantly lower levels of distress prior to radiotherapy planning (first follow-up [F1]) compared with usual care.

### Secondary hypotheses

1. After controlling for baseline distress, intervention patients will report significantly lower levels of distress at treatment commencement (second follow-up [F2]) compared with usual care.
2. After controlling for baseline between-group differences on the target outcome, intervention group participants will report significantly lower levels of anxiety, depression, concerns about radiotherapy and significantly higher levels of knowledge of radiotherapy and preparedness at F1 and F2 compared with usual care.

## Methods

### Trial design

A multiple**-**baseline methodology (repeated measures design) was used. Multiple baseline designs are appropriate when testing interventions designed to change health professionals’ behaviour^34^. This methodology facilitates systematic comparisons of pre and post-intervention measures while controlling for other factors.

The trial was registered on the Australian and New Zealand Clinical Trials Registry: ACTRN12611001000998. Ethics approval was gained from Curtin University (HR123/2011) and the participating sites.

### Intervention content

The RT Prepare intervention consisted of face-to-face consultation with a RT (1) prior to planning and (2) prior to treatment^35^. During consultations, RTs provided sensory and procedural information, assessed the psychosocial needs of patients and coached the patient in anxiety reduction strategies. RTs used a checklist to guide discussion. The consultations were tailored based on individuals’ information and support needs (Supplement 1).

### Training

Training for RTs consisted of a: (1) communication skills workshop on eliciting and responding to emotional cues; and (2) radiotherapy specific workshop on sensory and preparatory information.

### Settings

Participating sites were in Perth, Adelaide and Melbourne, Australia. Recruitment started in July 2012, ceasing in December 2015. The three sites were randomised with respect to the order in which the intervention commenced. Training was provided after 12 months at Site 2, 18 months at Site 1 and 24 months at Site 3. Transparent Reporting of Evaluations with Non-Randomised Designs (TREND) guidelines were followed^36^.

### Sample recruitment

Eligibility criteria: diagnosed with early breast cancer; referred for curative radiotherapy ( ≥ 50 Gy equivalent); had not commenced planning or treatment; planning scheduled at least 2 days after recruitment; no cognitive impairments or psychiatric illnesses; and able to communicate in English. Women receiving chemotherapy and radiotherapy and those referred for radiotherapy alone were included. Patients were recruited following their first radiation oncologist appointment. Informed consent was gained from all participants prior to completing baseline surveys.

### Measures

The primary outcome measure was psychological distress using the total score for the HADs-T^32,33^.

The secondary outcome measures were: anxiety using the seven anxiety items within the HADs-A; depression using the seven depression items within the HADs-D; concerns about radiotherapy using the Concerns about RT scale^37^; patient knowledge of radiotherapy using the Knowledge of RT scale (including knowledge of planning and treatment subscales); patient preparedness (including procedural and sensory-psychological concerns) using the Cancer Treatment Survey^38^. All measures had established psychometric properties and had been used with the target population.

Participants completed eight study-specific single Likert scale items, on preparation for, and anxiety about, planning procedures and receiving radiotherapy.

### Survey timepoints

**Fig. 1:**
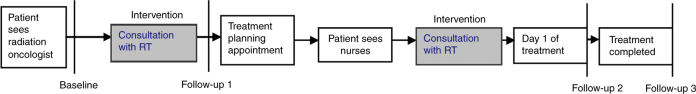
Timing of intervention delivery and survey distribution

Participants completed baseline surveys after seeing their radiation oncologist and prior to attending their planning appointment; F1 was completed in the department prior to their planning appointment (for intervention participants this was after intervention delivery and prior to planning); F2 was completed within 24–48 h of the patient’s first treatment; follow-up three (F3) was completed within a week of treatment completion (Fig. 1). The time between Baseline and F1 was dependent on whether patients were receiving chemotherapy and department waiting lists. The average time between F1 and F2 was 1 week, and between F2 and F3 was 6–7 weeks.

All measures were administered at baseline and F1. At F2 the patient knowledge of RT subscale relating to planning was not administered because it was no longer relevant. At F3 the patient knowledge questionnaire was not administered or the single item questions relating to procedures because patients had completed treatment.

### Quality assurance

All intervention consultations were digitally recorded. Fifteen percent were randomly selected and analysed using a quality assurance protocol^31^. Workshop facilitators reviewed the first 10% to provide feedback to RTs during follow-up workshops at each site^39^.

Fifteen percent of usual care appointments were digitally recorded and analysed (Supplement 1).

### Sample size and power calculation

Our pilot data (*n* = 123) indicated a 0.4 SD difference between usual care and intervention groups in psychological distress using the HADs total score. At a two-tailed alpha-level of 0.05, a sample size of 200 patients (100 in each group), was required for 80% power to detect between-group differences on the primary outcome of 0.4 SD^40^. This equated to 67 patients per site. Adjusting for a design effect of 1.66 (assuming an ICC = 0.01 and 67 patients for each of the three sites), the revised sample size was 332 patients (166 each in usual care and intervention groups, 111 per site).

### Statistical analyses

A series of Generalised Linear Mixed Models (GLMMs) were applied, one for each outcome measure, to test the significance of the between-group comparisons at each post-intervention assessment controlling for between-group differences at baseline and intra-site dependencies using the SPSS (Version 24) GENLINMIXED procedure. Each GLMM included the baseline scores as a covariate; participant as a random effect; group and site as nominal fixed effects; time as an ordinal fixed effect; and the interactions among these variables. Inclusion of the baseline scores in the model ameliorated potential effects from participant attrition across time. For secondary outcome measures where two subscales within the same scale were tested, the between-group difference for each subscale was evaluated at a Bonferroni-adjusted alpha-level of .025.

A reliable change (RC) score was computed for each participant to determine whether a participant’s psychological distress score improved (or deteriorated) across time^41^. The RC score can be interpreted as the degree to which the person changes on the outcome variable between timepoints divided by the standard error of difference between timepoints. Absolute values of the RC score > 1.96^42^ reflect a RC over time rather than fluctuations of an imprecise measuring instrument^41,43^.

### Intervention costs

Intervention delivery costs were estimated in two ways. Within-trial costs were estimated including (1) RTs’ time in delivering the two intervention consultations, based on hourly rates for RTs (including oncosts), (2) RTs time attending training workshops and (3) workshop facilitators and actor’s time required to prepare and run the workshops. Continuing costs per patient incurred if the intervention were maintained and available to all eligible patients at the three sites were estimated. These excluded (2) and (3) above as workshops would not need to be run again for all staff. Continuing costs included (1) above, (2) annual follow-up training to RTs to maintain skills (a single 2-hour workshop at each site), and (3) training of new RTs at any of the sites. Total training costs (2 and 3) were divided by the total eligible patient numbers across the three sites in 2015. All costs are in 2015 Australian dollars with 2015 US dollars in parentheses.

## Results

**Fig. 2:**
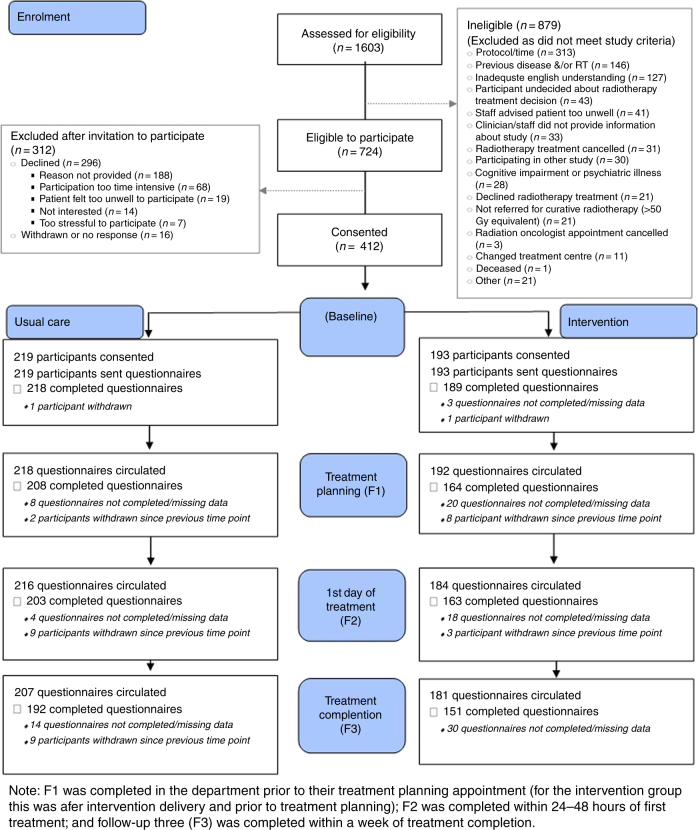
Study flow diagram

Figure 2 summarises recruitment and participant numbers. There was no significant difference for women who declined participating in relation to age (*p* > 0.05). Additional demographics for non-participants were not collected.

The attrition rates were usual care: 12%; intervention: 21%. Drop-outs from the intervention group did not report higher distress at baseline than drop-outs from the control group (*p* = 0.52).

### Participant details

Table 1 summarises participant details. No significant differences were found between usual care and intervention groups for any demographic variables.

**Table 1:** Demographic descriptors of control and intervention groups

	Usual care (*n* = 218)	Intervention (*n* = 190)	Tests of between-group differences
	*M* (*SD*)	*M* (*SD*)	
Age	55. 9 (10.5)	57.9 (10.5)	*t*(395) = 1.93, *p* = 0.055
	*n* (%)	*n* (%)	
Site			*χ*^2^(2) = 2.91, *p* = 0.233
Site 1 (Adelaide)	65 (29.8)	56 (29.5)	
Site 2 (Melbourne)	100 (45.9)	100 (52.6)	
Site 3 (Perth)	53 (24.3)	34 (17.9)	
Marital status			*χ*^2^(1) = 0.18, *p* = 0.669
In a relationship	149 (68.3)	124 (65.3)	
Not in a relationship	68 (31.2)	62 (32.6)	
Missing	1 (0.5)	4 (2.1)	
Country of birth			*χ*^2^(7) = 8.35, *p* = 0.303
Australia	130 (59.6)	132 (69.5)	
UK	44 (20.2)	22 (11.6)	
Europe	10 (4.6)	12 (6.3)	
New Zealand	8 (3.7)	6 (3.2)	
Asia	7 (3.2)	3 (1.6)	
India/ Sri Lanka	5 (2.3)	3 (1.6)	
Africa/ South Africa	5 (2.3)	5 (2.6)	
Other	7 (3.2)	5 (2.6)	
Missing	2 (0.9)	2 (1.1)	
Australian Citizen or permanent resident?			*χ*^2^(2) = 5.21, *p* = 0.074
Citizen	191 (87.6)	177 (93.2)	
Permanent resident	22 (10.1)	8 (4.2)	
Neither	3 (1.4)	2 (1.0)	
Missing	2 (0.9)	3 (1.6)	
Speaks English at home			*χ*^2^(1) = 1.07, *p* = 0.301
Yes	206 (94.5)	182 (95.8)	
No	10 (4.6)	5 (2.6)	
Missing	2 (0.9)	3 (1.6)	
Education			*χ*^2^(2) = 1.31, *p* = 0.519
High School or lower	107 (49.1)	81 (42.6)	
Technical and Further Education (TAFE)	53 (24.3)	48 (25.3)	
University	57 (26.1)	56 (29.5)	
Other	0 (0.0)	2 (1.0)	
Missing	1 (0.5)	3 (1.6)	
Employment			*χ*^2^(1) = 1.07, *p* = 0.485
Employed/studying	127 (58.3)	104 (54.7)	
Unemployed/other	88 (40.3)	83 (43.7)	
Missing	3 (1.4%)	3 (1.6)	
Type of surgery^a^			n/a
Lumpectomy/partial mastectomy	179 (82.1)	163 (85.8)	
Mastectomy	40 (18.3)	26 (13.7)	
Breast reconstruction	6 (2.8)	5 (2.6)	
Other	3 (1.4)	4 (2.1)	
Chemotherapy			*χ*^2^(1) = 0.73, *p* = 0.394
Yes	107 (49.1)	102 (53.7)	
No	107 (49.1)	86 (45.3)	
Missing	4 (1.8)	2 (1.0)	
Other health conditions			*χ*^2^(1) = 0.40, *p* = 0.529
Yes	112 (51.6)	104 (54.7)	
No	105 (48.4)	86 (45.3)	
Patient understanding of radiotherapy prior to cancer diagnosis (Likert Scale - 1–9)			*χ*^2^(2) = 2.84, *p* = 0.241
No understanding (1)	56 (25.7)	36 (18.9)	
Some (2–3)	61 (28.0)	53 (27.9)	
Moderate or better ( ≥ 4)	99 (45.4)	98 (51.6)	
Missing	2 (0.9)	3 (1.6)	

^a^ Multiple responses possible

### Psychological distress

**Fig. 3:**
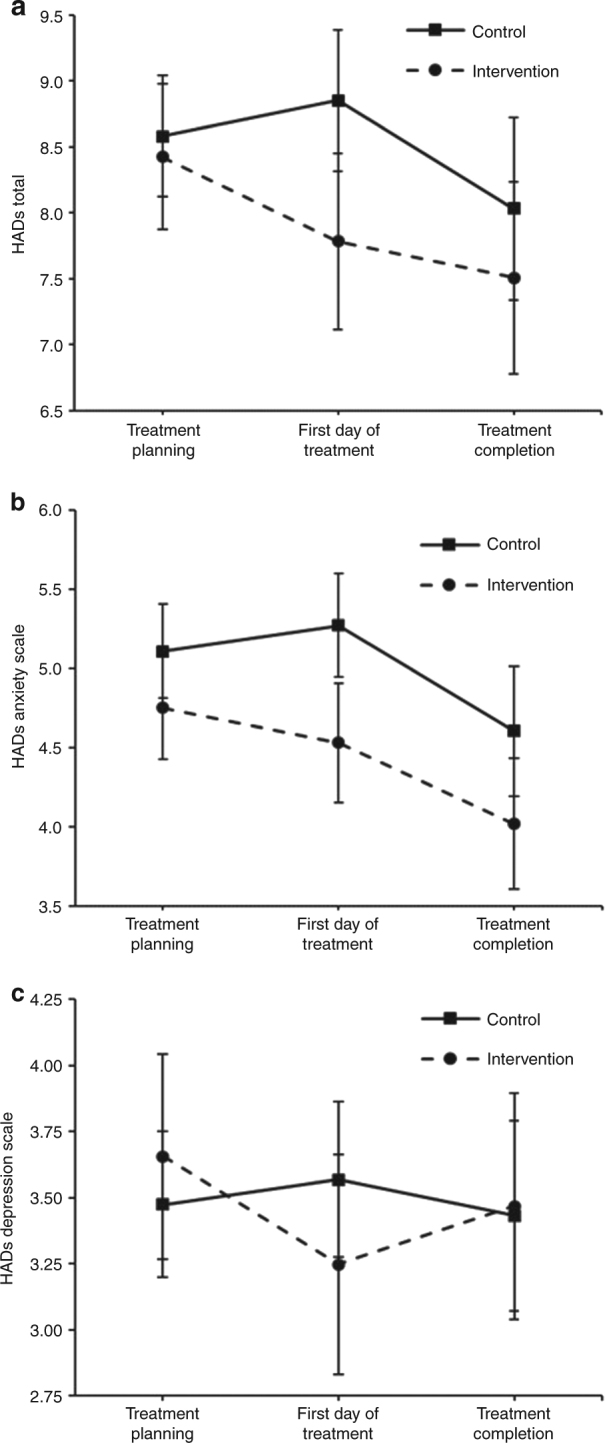
Comparison of control and intervention scores for HADs total **a** HADs anxiety **b**, and HADs depression **c**

The mean HADs-T (adjusted for between-group differences on the outcome at baseline), and standard errors are reported in Table 2. The intervention group reported significantly lower HADs-T at F2 (*p* = 0.01) compared with the control group, but not at F1 or F3 (Fig. 3a, Table 3).

**Table 2:** Raw baseline means and adjusted post-test means^a^, standard errors, and 95% confidence intervals for HADs Scale

Outcome	Baseline			Treatment planning (F1)			First day of treatment (F2)			Treatment completion (F3)		
	Mean	SE	95% CI	Mean	SE	95% CI	Mean	SE	95% CI	Mean	SE	95% CI
Intervention												
HADs total	9.15	0.46	8.25–10.74	8.43	0.28	7.87–8.98	7.79	0.34	7.12–8.45	7.51	0.37	6.78–8.24
HADs anxiety	5.46	0.28	4.92–6.01	4.75	0.17	4.43–5.08	4.53	0.19	4.15–4.91	4.02	0.21	3.60–4.43
HADs depression	3.68	0.23	3.24–4.13	3.66	0.20	3.27–4.04	3.25	0.21	2.83–3.66	3.47	0.22	3.04–3.90
Control												
HADs total	9.76	0.50	8.79–10.74	8.58	0.23	8.12–9.04	8.85	0.27	8.32–9.39	8.03	0.35	7.34–8.72
HADs anxiety	5.81	0.29	5.24–6.37	5.11	0.15	4.81–5.41	5.27	0.17	4.95–5.60	4.60	0.21	4.20–5.01
HADs depression	3.96	0.26	3.46–4.46	3.47	0.14	3.20–3.75	3.57	0.15	3.27–3.86	3.43	0.18	3.07–3.79

^a^ Post-test means were adjusted for between-group differences on the outcomes at baseline. F1: follow-up 1; F2: follow-up 2; F3; follow-up 3

**Table 3:** Results from GLMM regression models testing between-group effects

Outcome	Prior to treatment planning (F1)				Treatment commencement (F2)				Treatment completion (F3)			
	Estimated mean difference	95% CI	*p*	Cohen’s d	Estimated mean difference	95% CI	*p*	Cohen’s d	Estimated mean difference	(95% CI)	p	Cohen’s *d*
Pyschological distress	0.16	−0.56–0.87	0.67	0.05	1.07	0.21–1.93	0.01*	0.16	0.53	−0.47–1.52	0.30	0.08
HADs total												
HADs anxiety	0.36	−0.08–0.80	0.11	0.09	0.74	0.25–1.24	0.003*	0.18	0.59	0.01–1.17	0.048*	0.15
HADs depression	−0.18	−0.66–0.29	0.45	0.05	0.32	−0.18–0.83	0.22	0.09	−0.037	−0.60–0.52	0.90	0.01
Concerns about RT	0.60	0.32–0.88	< 0.001^†^	0.27	0.33	0.07–0.60	0.01*	0.15	N.A.	-	-	-
Patient knowledge of RT	−3.25	−3.74 to −2.76	< 0.001^†^^	1.16	N.A.	–	–	–	N.A.	–	–	–
Subscale: planning												
Subscale: treatment	−3.23	−3.86 to −2.60	< 0.001^†^^	0.90	−1.36	−2.16 to −0.56	< 0.001^†^^	0.60	N.A.	-	-	-
Patient preparedness	0.44	0.26–0.62	< 0.001^†^^	0.43	0.33	0.15–0.52	< 0.001^†^^	0.32	0.15	−0.04–0.35	0.13	0.15
Subscale: procedural concerns												
Subscale: sensory- psychological concerns	0.33	0.19–0.48	< 0.001^†^^	0.35	0.15	−0.04–0.34	0.12	0.16	0.11	−0.04–0.26	0.15	0.12
Procedures: Preparation and anxiety	−1.82	−2.16 to −1.47	< 0.001†	0.96	−0.71	−1.02 to −0.39	< 0.001†	0.54	N.A.	–	–	–
How prepared do you currently feel for the treatment (planning) procedure?												
I know what is going to happen during the appointment.	−2.43	−2.77 to −2.09	< 0.001†	1.21	−0.81	−1.13 to −0.49	< 0.001†	0.56	N.A.	–	–	–
How anxious do you feel about the procedure?	0.44	0.048–0.84	0.03*	0.21	0.35	−0.10–0.80	0.13	0.16	N.A.	–	–	–
Treatment: preparation and anxiety	−1.43	−1.72 to −1.15	< 0.001^†^	0.24	−0.42	−0.73 to −0.11	0.009*	0.19	N.A.	–	–	–
How much understanding do you currently have of radiotherapy?												
How anxious do you feel about receiving radiotherapy?	0.52	0.12–0.92	0.01*	0.26	0.17	−0.30–0.65	0.47	0.09	N.A.	–	–	–
How prepared do you currently feel to receive radiotherapy?	−1.56	−1.85 to −1.26	< 0.001^†^	0.74	−0.51	−0.80 to −0.22	< 0.001^†^	0.24	N.A.	–	–	–
I know what is going to happen during my treatment	−1.69	−2.05 to −1.34	< 0.001^†^	0.87	−0.62	−0.96 to −0.28	< 0.001^†^	0.32	N.A.	–	–	–
Did the information that you received to date meet your expectations?	−1.59	−1.88 to −1.29	< 0.001^†^	0.88	0.41	−0.69 to −0.13	0.004*	0.23	N.A.	–	–	–

*GLMM*: generalised linear mixed models. Estimated mean difference measured between intervention and control. * p < 0.05, † *p* < 0.001

^ Significant at the Bonferroni-adjusted alpha-level of .025. N.A.: not administered F1: follow-up 1; F2: follow-up 2; F3; follow-up 3

### Anxiety and depression

The intervention group reported significantly lower levels of anxiety at F2 (*p* = 0.003) and F3 (*p* = 0.048) compared with the control group (Fig. 3b, Table 3). There were no significant differences for depression at any follow-up timepoints (*p* > 0.05) (Fig. 3c, Table 3).

### RC analysis

For HADs-T there was a prevention effect. Only 1.3% (*n* = 2) of intervention participants showed reliable increases in HADs-T from baseline to treatment completion, whereas 7.0% (*n* = 13) of the control group became reliably more distressed at treatment completion (*p* = .046). For anxiety, the intervention also had a prevention effect. None of the intervention participants showed reliable increases in anxiety from baseline to treatment commencement, whereas 3.6% (*n* = 7) of the control group became reliably more anxious at treatment commencement (*p* = .031). Only 0.7% (*n* = 1) of the intervention group showed a reliable increase in anxiety from baseline to treatment completion; whereas 5.4% (*n* = 10) of the control group became reliably more anxious at treatment completion, however the between-group difference was not significant (*p* *=* .057). For depression, there was no significant difference (*p* > 0.05) at any timepoint between the two groups in terms of the proportion of participants showing RC (either improvement or deterioration).

### Concerns about radiotherapy

The intervention group reported significantly lower concerns about radiotherapy than the control group at F1 (*p* < 0.001) and F2 (*p* = 0.01).

### Knowledge of radiotherapy

Compared with the control group, the intervention group reported significantly higher knowledge of planning at F1 (*p* < 0.001, Table 3). In addition, for the treatment subscale, the intervention group reported significantly higher knowledge regarding treatment at F1 and F2 compared to the control group (*p* < 0.001).

### Patient preparedness

The intervention group reported significantly lower scores on the procedural concerns subscale of the Cancer Treatment Scale at F1 (*p* < 0.001) and F2 (*p* < 0.001), but not at F3 (*p* = 0.13). For the Sensory-Psychological Concerns subscale, the intervention group reported significantly lower scores at F1 (*p* < 0.001), whereas no significant differences were found at F2 (*p* = 0.12) or F3 (*p* = 0.15).

Table 3 demonstrates where significant differences were found on single-items relating to preparation, knowledge and anxiety about planning and treatment procedures.

### Intervention costs

Average times for delivering the planning consultation were: 13 min (SD = 5.2), 18 min (SD = 7.2) and 21 min (SD = 6.1) at Sites 1, 2, 3 respectively. Average times for delivering the treatment consultation were: 16 min (SD = 6.4), 17 min (SD = 5.5) and 19 min (SD = 6.1). Thus, mean durations for the two consultations combined were 29, 36 and 41 min at Sites 1, 2 and 3. Multiplying these by RTs hourly rates (including oncosts) resulted in a weighted average cost for consultations of AU$25 (US$19). Training workshops cost an estimated AU$25,425 (US$19,175); distributing this across all patients in the intervention group in addition to the consultation costs resulted in a mean within-trial cost of AU$159 (US$120).

Costs of annual workshops to provide updated training to all RTs at three sites were estimated at AU$6,151 (US$4639), whereas costs of annual workshops to train new staff were estimated at AU$8155 (US$6150), a total annual training cost of AU$14,305 (US$10,788). Distributing this across all eligible patients in 2015 (total of 1413 across all sites) in addition to the average consultation cost above resulted in a mean continuing cost of AU$35(US$26) per patient.

## DISCUSSION

Agreed standards for delivery of radiotherapy preparatory education are lacking^6,44^. Although patients are provided with education by their radiation oncologist at initial consultations, their information needs remain high before treatment^45^. RTs are well positioned to educate and support patients prior to treatment given their direct involvement in treatment delivery.

Psychological interventions using repeated session of cognitive-behavioural therapy and hypnosis^46,47^, patient diary led therapy^48^ and mindfulness-based stress reduction programmes^49^ resulted in significant improvements (with moderate to large effect sizes) in patients’ mood, distress levels and other psychosocial outcomes during and after radiotherapy. However, these studies were not focused on treatment preparation, were delivered outside of radiotherapy by clinical psychologists, psychiatrists or masters or doctoral students with appropriate training, and some of them required patients to participate in a series of additional appointments.

Previous studies testing information resources in radiotherapy (videos) failed to demonstrate significant reduction in patient anxiety prior to treatment^16–19^. Only one RCT (*n* = 220) reported significant decreases in anxiety and depression 3 weeks after commencing treatment using an educational video compared to usual care^18^. Group education was trialled in two underpowered studies, both concluding that anxiety was decreased following group education^20,21^.

Dong et al.^50^ reported state-trait anxiety was decreased when one-on-one pre-treatment education sessions were provided (*n* = 56). Aranda et al.’s^26^ RCT (*n* = 192) significantly reduced anxiety and sensory-psychological and procedural concerns following one-on-one chemotherapy education. In this study, compared with usual care, RT Prepare demonstrated reductions in anxiety and overall distress prior to treatment. This reduction in anxiety is critical to help patients deal with an unfamiliar environment, proceed and cope with treatment, and manage side effects^51^.

Appropriate communication that focuses on patients’ needs, is pitched at the patients’ level of understanding and provides emotional support is required by patients prior to commencing treatment^7^. The Aranda et al.^26^ and current study were based on Level I evidence on preparing patients for threatening medical procedures^22–24^, providing health professionals with communication skills training focusing on eliciting and responding to emotional cues^28^, and coaching patients in anxiety reduction techniques^27^. Both interventions provided evidence-based, structured consultations, covered a breadth of information, and tailored to individuals’ needs. To ensure protocol adherence, audit and feedback processes were applied.

The current study demonstrates that the role of RTs can be extended from a technological focus to providing patients with information and support prior to planning and treatment. Compared to usual care, RT Prepare significantly improved patient knowledge, reduced patient concerns about radiotherapy and improved patient preparedness prior to planning and treatment. Increasing patient preparedness has implications for efficiency, accuracy and adherence during treatment, all of which are essential for safe and effective radiotherapy delivery^13^. Findings reflect those of Merchant et al.^52^ who highlighted the need to redesign radiotherapy environments and implement changes in workforce culture to enable RTs to support patients. This change in roles and skill development for RTs would also reduce demands on other health professionals and hospital services.

The feasibility and cost effectiveness of training RTs to deliver preparatory education is evident. The training was effective and delivered in a single-day compared to a 38-hour training programme provided in Belgium. RT Prepare was inexpensive to deliver in comparison with costs reported elsewhere for psychosocial interventions tested with cancer survivors^53^.

### Limitations

This study used a multiple-baseline design because an RCT or cluster-randomised trial were not feasible to test an intervention of this kind. Given the temporal separation of recruitment to usual care and intervention groups, changes in health care could have impacted our findings. However, randomisation of the commencement times of the intervention would mitigate such impacts and no practice changes likely to influence the study outcomes were identified.

The results of this study may have been strengthened by the use of an “active control group”, such as providing professional attention^54^. However, as the intervention we were testing was not previously established or demonstrated to have an effect it was appropriate to first determine whether there was a difference between the intervention and usual care as delivered^55^. Further research comparing usual care, an active control and the intervention might be appropriate.

Because we experienced difficulty in recruiting patients (57% of those eligible) and participants were self-selected, the generalisability of the results may be compromised as the sample may not be representative of all women diagnosed with early breast cancer and referred for radiotherapy. Although retention rates were high (83% of baseline at F3), attrition, may also have biased findings. Differences between intervention and control groups would have been partially controlled for by the inclusion of baseline outcome scores in the GLMM modelling, ameliorating this potential source of bias. Differences in participation and intervention delivery between sites were also accounted for in the GLMM analyses.

Patients completed F1 following the first intervention and prior to the planning appointment. However, the lack of differences on the depression scores negates the possibility that social desirability bias is the explanation for the lower anxiety scores at follow-up (i.e., participants reporting less anxiety than they were feeling).

The intervention was effective in increasing patient preparedness in relation to procedural concerns and sensory concerns at F1 and procedural concerns at F2. It is not unexpected that no difference was found between the intervention and usual care group for sensory-psychological concerns at F2 because once patients received information about how this procedure feels and participated in the treatment planning appointment they would have had less concerns about this. Furthermore, as the questionnaire for F2 was completed after receiving their first treatment both groups had experienced how treatment feels, hence further reducing any concerns in this area.

The effect sizes on the HADs-T and HADs-A at follow-up, although statistically significant, are relatively small^56^. These effect sizes are similar to the small sizes reported by Aranda et al.^26^. Other studies testing change in anxiety following radiation therapy education do not report effect sizes. Effect size is one criteria for determining whether an intervention is clinically significant. Other criteria, relevant to RT Prepare, are that the intervention affected other outcomes of interest to patients (concerns, knowledge of and preparedness for treatment); did more good than harm; and was affordable^57^. To increase the intervention effect in reducing patient anxiety, future trials might provide additional follow-up training and individual feedback to RTs to enhance the support provided to patients prior to treatment.

## CONCLUSION

This is the first study internationally to trial, using a multiple-baseline design, an inexpensive intervention delivered by RTs, which addresses patients’ information and support needs and reduces levels of anxiety prior to treatment. The potential exists to extend the role of RTs by considering workforce redesign to improve how patients are routinely prepared for treatment. Additional testing of this intervention should explore whether the effect of this intervention can be increased by providing additional follow-up training and individual feedback to RTs to enhance the support provided to patients prior to treatment. Future work needs to focus on testing this intervention with additional participants, other patient groups and exploring translation into practice. Because patients self-selected to participate in this study, further implementation testing is warranted to see whether the intervention is beneficial for a wider population of participants.

## Electronic supplementary material

Supplement 1

## Availability of data and material

Please contact the corresponding author.
