# Supplementary material for: RT Prepare: a radiation therapist-delivered intervention reduces psychological distress in women with breast cancer referred for radiotherapy
Source: Br J Cancer. 2018 Jun 1;118(12):1549–58. doi: 10.1038/s41416-018-0112-z (PMC6008448; doi:10.1038/s41416-018-0112-z)
Supplement: Supplementary file 1 — Supplement 1 [file 41416_2018_112_MOESM1_ESM.docx]

## Supplement 1 – Description of the intervention, usual care and quality assurance

## Intervention content

The tailored educational intervention package consisted of a face-to-face consultation with a radiation therapist (RT) in a private room (1) prior to treatment planning and (2) prior to ﬁrst day of treatment. During the consultations, the RT provided sensory and procedural information, assessed the psychosocial needs of patients and coached the patient in anxiety reduction strategies when applicable. Sensory information focused on describing to patients how they were likely to feel before, during and after the procedure; procedural information focuses on describing the procedure such as what the RTs would be doing, measurements that would be taken and positioning. RTs were provided with a checklist to guide them on topics to discuss at each time point. The consultations were tailored to individual patient needs depending on what information and support needs were identified by RTs during the consultations. During the ﬁrst consultation, prior to treatment planning, RTs focused on discussing topics relating to treatment planning and identified patients’ emotions, specifically fears and anxieties ^1, 2^. During the second consultation, prior to treatment, RTs discussed information needs relating to treatment and what to expect, and identified patients’ fears and anxieties ^1^. During communication skills training RTs learnt how to elicit the following emotional cues: anxiety, depression, distress and anger and respond appropriately ^3^. RTs were trained to use the following strategies for understanding information needs and detecting anxiety and distress: active listening and using open questions, responding to patients’ emotional cues and adopting a patient-centred communication style ^4^. If patients were experiencing anxiety or distress, the RTs were trained to use strategies to find out more about the patient’s feelings, provide advice about anxiety reduction techniques and refer for psychological support if required.

**Intervention Fidelity**

All intervention consultations were digitally recorded. Fifteen percent of recordings were randomly selected and analysed using a quality assurance protocol to ensure that intervention delivery was consistent with the protocol ^5^. The quality assurance protocol included information about the procedures as well as tasks relating to eliciting and responding to emotional cues ^1^. Table 1 summarises areas that were assessed. Tape recordings were assessed by a clinical psychologist and a Final Year Radiation Therapy Graduate Entry Masters Student. The assessment scores recorded were within 5% after the student received training and scores were discussed between the two assessors.

**Table 1 –Summary of Components included in Quality Assurance Protocol**

| **Component** | **Summary** |
| --- | --- |
| **Setting the Scene** | - Develops a rapport with the patient - Discusses treatment patient has received to date - Explains what is going to happen - Elicits patients concerns and reactions - Discusses what information patient has received - Checks whether patient has consented for treatment - Describes role of radiation therapist - Explores what patient knows about radiation therapy - Explores how patient is feeling about procedures - Determines if patient has any psychosocial issues that are going to make it difficult to complete planning appointment and/or proceed with treatment - Determines if patient has any fears and anxieties relating to planning and treatment procedures |
| **Explains Procedure** | - Duration of appointment - Why procedure is necessary - **Provides Sensory information:** Describes what procedure is going to feel like (including skin sensations, smells, sounds, sights, level of comfort) - Explains patient will be left alone in room and what this might feel like, highlighting that RTs have video cameras to watch them and that they can call the RTs if necessary - Determine if patient is able to lie in position and keep still - Determine physical limitations for individual patient - **Provides Procedural information** about measurements, land marking, tattoos - Area to be included within treatment field - Equipment used - Describes positioning and importance of keeping still - Asks whether the patient has concerns - Discusses possible side effects - Provides opportunity to ask questions - Ensures patient has ongoing involvement in consultation |
| **Patient Understanding** | - Enables patient to explain their perspective fully - Phrases questions simply and clearly - Seeks clarification of words used by patient as appropriate - Checks that patient understands what has been said - Uses clear and understandable language - Maintains friendly but professional relationship with patient - Anticipates and flags close of consultation - Acknowledges that much information has been given and concerns may arise after the consultation - Repeats and summarises important information |
| **Location and timing** | - Education completed in appropriate location - Education completed without "feeling rushed" |
| **Active listening techniques** | - Minimal prompts used - No interruptions - Paraphrasing responses - Summarise |
| **Responds empathetically to patient emotions** | - Identifies core message - Takes time to think and respond - Frequent short responses to convey empathy - Checks back that feelings have been interpreted correctly |
| **Potential emotional responses and appropriate responses** | **Responds to Anxiety**   - Asks scanning questions - Acknowledges and normalises anxiety - Explores main source of anxiety - Checks informational needs are met - Refers if warranted   **Responds to Depression**   - Asks scanning questions - Indicates concern about the patient - Indicates that depression is common and important - Suggests that patient be referred to a specialist   **Responds appropriately to Distress**   - Sits quietly through the tears - Normalises the experience - Asks "Do you want to talk about this now?" - Explores social support - Offers professional support   **Responds to Anger**   - Stays calm - Listens actively-focuses on the issue - Acknowledges anger and explores the reasons for it - Focuses on the person's needs, not their manner or words - Brainstorms options and offers help |
| **Blocking behaviours** | - Interrupting - Monopolising - Switched off and part listening - Disruptions - Mind reading - Changing the subject - Intimidation - False reassurance and placating - Judging - Interrogating |

Please note that this is a summary of the components and does not include all areas covered in the Quality Assurance Assessment.

## Usual Care

## Usual care consisted of patients being provided with information by their radiation oncologist, radiation oncology nurse and RTs at the following timepoints: initial radiation oncologist consult, treatment planning appointment, and on the first day of treatment. Research assistants in each of the sites digitally recorded 15% of usual care appointments with RTs in order to document the care provided. Usual care recordings were analysed using the same criteria and Quality Assurance protocol used to assess the intervention delivery. Overall, during usual care RTs delivered information quickly prior to commencing the procedures. At treatment planning the focus was for the patient to have the CT procedure completed and information delivery was focused on this. At treatment commencement RTs delivered a first day chat where more information was provided about treatment and what would be involved.

## Our previous work has demonstrated that information provision within radiotherapy departments is inconsistent and varies in terms of who provides information, when information is provided and how information is provided^6^. There was also variation found in usual care delivery between the three participating sites in this study. However, this variation was accounted for in the data analysis.

**Comparison between usual care and intervention**

In comparison to intervention participants, usual care participants were less likely to receive emotional support, detailed information about the procedures or have the opportunity to ask questions or discuss their concerns about radiotherapy. Furthermore, usual care was delivered in a shorter time frame on average than the intervention where RTs had time to sit down with the patients. On average the intervention consultations at both time points took approximately 17 mins, compared to 7.5 minutes for usual care. At the CT appointment, the overall percent correct score for the QA assessment for RTs delivering the intervention was higher (Median=62.99) than RTs who provided usual care (Median=39.61) *U* = 301.00, *z* = 4.38, p<0.001, r = 0.72. On the first day of treatment, the overall percent correct score of RTs who delivered the intervention was higher for the QA assessment (Median=70.11) than RTs delivering care to patients in the usual care group (Median=37.93) *U* = 276.00, *z* = 4.80, p<0.001, r = 0.81.

RTs who delivered the intervention scored higher percentages in the QA assessment than RTs delivering usual care at both time points in the following areas: ‘setting the scene and explains procedure’, ‘responding empathetically to patient emotions’ and ‘patient understanding’ (Table 2).

Table 2 – Average percentage scores RTs achieved delivering the intervention or usual care for the main three components of the QA assessment

|  | **Setting the scene & explain procedure** | **Responds empathetically to patient emotions** | **Patient understanding** |
| --- | --- | --- | --- |
| Intervention - Planning | 60.6% | 28.5% | 61.2% |
| Usual care – Planning | 31.9% | 9.5% | 45.2% |
| Intervention – Treatment | 73.2% | 41.3% | 68.9% |
| Usual care – Treatment | 30.5% | 2.4% | 37.7% |

RTs delivering the intervention were also assessed by the reviewers as being better equipped to elicit emotional cues (anxiety, depression, distress and anger) and respond appropriately.

**References**

1. Halkett GK, O'Connor M, Aranda S, Jefford M, Shaw T, York D, et al. Pilot randomised controlled trial of a radiation therapist-led educational intervention for breast cancer patients prior to commencing radiotherapy. *Support Care Cancer*. 2013 Jun;21(6):1725-33.

2. Halkett GK, Schofield P, O'Connor M, York D, Jefford M, Jiwa M, et al. Development and pilot testing of a radiation therapist-led educational intervention for breast cancer patients prior to commencing radiotherapy. *Asia Pac J Clin Oncol*. 2012 Sep;8(3):e1-8.

3. Butow P, Cockburn J, Girgis A, Bowman D, Schofield P, D'Este C, et al. Increasing oncologists' skills in eliciting and responding to emotional cues: evaluation of a communication skills training program. *Psychooncology*. 2008;17:209-18.

4. Ryan H, Schofield P, Cockburn J, Nutow P, Tattersall M, Turner J, et al. How to recognise and manage psychological distress in cancer patients. *Eur J Cancer Care*. 2005;14:7-15.

5. Aranda S, Jefford M, Yates P, Gough K, Seymour J, Francis P, et al. Impact of a novel nurse-led prechemotherapy education intervention (ChemoEd) on patient distress...results from a randomised controlled trial. *Ann Oncol*. 2012 Jan;23(1):222-31.

6. Halkett GK, Short M, Kristjanson LJ. How do radiation oncology health professionals inform breast cancer patients about the medical and technical aspects of their treatment? *Radiotherapy and oncology : journal of the European Society for Therapeutic Radiology and Oncology*. 2009 Jan;90(1):153-9.
